# Effective Augmentation of Creativity-Involving Productivity Consequent to Spontaneous Selectivity in Knowledge Acquisition

**DOI:** 10.3389/fpsyg.2019.00600

**Published:** 2019-03-28

**Authors:** Hiroki Kurashige, Yuichi Yamashita, Takashi Hanakawa, Manabu Honda

**Affiliations:** ^1^Graduate School of Informatics and Engineering, The University of Electro-Communications, Tokyo, Japan; ^2^National Center of Neurology and Psychiatry, National Institute of Neuroscience, Tokyo, Japan; ^3^Integrative Brain Imaging Center, National Center of Neurology and Psychiatry, Tokyo, Japan

**Keywords:** knowledge acquisition, learning, creativity-involving productivity, text composition, human behavioral study, neural network simulation

## Abstract

The results of many studies have suggested that we actively select information from the environment. However, the functional consequences of such selectivity in knowledge acquisition remain unclear, even though it is a vital factor in determining the characteristics of our future knowledge and cognition. We hypothesized that spontaneous selectivity in knowledge acquisition results in effective augmentation of productivity, especially in creativity-demanding task. To test this, we conducted experiments in which subjects acquired novel compositional words during their rapid presentation, evaluated memory confidence rates for the acquired words, and then produced essays based on these words. First, in experiment 1, we showed that the level of confidence in the recognition memory for the words positively related with the length of the essays (a measure of creativity-involving productivity in quantity). Additionally, we found that the semantic distance from the essay to the components of the compositional word (a measure of creative-productivity in quality) was farther for the word with higher memory confidence than for the word with lower memory confidence, suggesting creative leaps when writing the former. While this result supported our hypothesis, it might also reflect better memory that was independent of spontaneous selection. Thus, in a different subject group, we conducted a similar experiment (experiment 2) in which two of the 20 compositional words were presented more often (five times per block) to force memorization. Again, consistent with our hypothesis, essays based on spontaneously memorized words (presented once per block) were significantly longer than those produced using the forcedly memorized words. Therefore, better memory *per se* did not explain the higher productivity. Instead, these results suggested that the higher creativity-involving productivity was consequent to spontaneous selectivity in the knowledge acquisition. Additionally, we propose a possible mechanism for the observed results based on the results of a neural network simulation. In this simulation, we found that novel information that was assigned to locations more easily accessible to the entire network was better assimilated and therefore selectively acquired. Based on this simulation, we moderately suggest that spontaneously acquired knowledge effectively confers productivity because it effectively activates large parts of the neural networks.

## Introduction

Knowledge acquisition is not a passive process. Our brains are not constantly assimilating flowing information but rather are actively selecting the information to acquire from the environment. In fact, compelling evidence gathered from psychology and cognitive neuroscience experiments supports the view of the active and selective acquisition of knowledge. Because of such selectivity, knowledge acquisition should make and remake our knowledge base, worldview, and entire cognitive ability in a special way. However, little is known of the functional *consequences* and underlying *mechanisms* of selective knowledge acquisition. In this study, we addressed these issues using human behavioral experiments and neural network simulations.

First, we will show experimental evidences to suggest that knowledge acquisition is selective and is governed by multiple factors that confer selectivity. One of the most influential factors is prior knowledge or pre-existing schema (van Kesteren et al., 2012; Brod et al., 2013; Ghosh and Gilboa, 2014). In general, information that is congruent with prior knowledge is more easily assimilated than incongruent information. Numerous studies have reported that items that are related to prior knowledge are more easily acquired, whether through daily life, academic learning, or laboratory experiments (Dooling and Lachman, 1971; Bransford and Johnson, 1972; Dooling and Mullet, 1973; van Kesteren et al., 2010a,b, 2013, 2014; van Buuren et al., 2014; Brod et al., 2015, 2016; Liu et al., 2017; Sommer, 2017). Subjects required to memorize pairs of items show better memory performances for congruent associations than for incongruent associations (van Kesteren et al., 2010b, 2013). One plausible reason is that the common knowledge constructed in daily life helps subjects assimilate novel, but commonsensical, pairs of items. Furthermore, the enhancing effects of prior knowledge on the acquisition of novel knowledge have been confirmed in more specialized fields. The effortful acquisition of academic facts facilitates further acquisition in related subjects (van Kesteren et al., 2014; Brod et al., 2016). Recently, we showed that prototypical neural representation that is arranged prior to experience plays a role in subsequent knowledge selection in a manner depending on prior knowledge (Kurashige et al., 2018).

Familiarity is also important for forming new knowledge. In associative learning, even if there is no common relationship between the items to be paired, familiarity for at least one item facilitates an association (Brod et al., 2016; Liu et al., 2017). Such enhancing effects of familiarity are also observed in the implicit learning of artificial grammar by exemplification (Scott and Dienes, 2010). In some cases, however, prior knowledge interferes with the acquisition of additional knowledge (Lipson, 1982; Alvermann et al., 1985; Kendeou and van den Broek, 2007; Sweegers et al., 2015).

Anticipation that the presented information will be used later is another important factor for successful knowledge acquisition, especially when sleep consolidation is involved. In one study, subjects informed about a retrieval test achieved better memory performance than uninformed subjects when the test was performed after sleep (Wilhelm et al., 2011). Another study found that subjects instructed that one-half of the encoded associations would be tested later and the other half would not be tested exhibited superior recall for the half they knew would be tested (anticipatory information) after sleep (van Dongen et al., 2012). In both studies, subjects that did not sleep between the presentation of the anticipatory information and testing did not show enhancement of learning. Similarly, anticipation for a rewarding test also enhanced sleep consolidation in motor skill learning (Fischer and Born, 2009).

The actual use or output of information also augments knowledge acquisition (Karpicke and Roediger, 2008; Carpenter, 2009; Pyc and Rawson, 2010; Roediger and Butler, 2011; Wing et al., 2013). For example, the importance of repeated output on tests has been suggested to promote the memory consolidation of foreign language vocabulary (Karpicke and Roediger, 2008). In that study, four conditions were compared. In the first condition, the items were repeatedly presented both in encoding and testing trials over the entire experiment. In the second condition, the items answered correctly once were dropped from further encoding trials. In the third condition, correctly answered items were dropped from further testing trials. In the fourth condition, correctly answered items were dropped from both the encoding and testing trials. Only in the conditions in which the items were dropped from the testing trials was memory performance impaired. Therefore, the actual output of items on tests is crucial for knowledge consolidation, which is rational because the items that were used should be considered more important than the items that were not used. Several studies suggest that such an effect may be due to the elaboration of the path to retrieval through the actual output (Carpenter, 2009; Pyc and Rawson, 2010).

Curiosity is also vital for knowledge acquisition. In our daily lives, we prefer to learn about things of which we are curious. Moreover, several studies have found that greater curiosity leads to better memory performance, and accompanying functional imaging has suggested that the activity of memory-related brain areas has beneficial effects on the learning of items arousing curiosity (Kang et al., 2009; Gruber et al., 2014).

Forgetting is another mechanism that indirectly contributes to selective knowledge acquisition. Several studies have suggested that forgetting occurs adaptively to prevent interferences in memory and reduce the cognitive demands of future tasks (Kuhl et al., 2007; Wimber et al., 2015). In typical experimental conditions, subjects were asked to associate both target and competitor with the same cue. Repetitive retrieval of the target caused subjects to forget the competitor. Strikingly, it decreased demands on cognitive control systems including the anterior cingulate cortex and the lateral prefrontal cortex (Kuhl et al., 2007).

Collectively, these findings strongly support the notion that we actively select the knowledge to acquire according to our needs. But what needs are satisfied and what functionality does such selectivity confer? Or, by selecting information, how are we going to sculpt our future knowledge, worldviews, and entire cognitive ability? To shed light on these questions, we must identify a cognitive ability that is augmented along with the spontaneous (selective) acquisition of knowledge. This implies that we need to identify a positive *consequence* of such a selectivity in knowledge.

Here, we hypothesized that spontaneous selectivity in knowledge acquisition results in effective augmentation of productivity. In other words, our hypothesis predicts that spontaneously selected knowledge effectively confers productivity on us compared to forcedly memorized knowledge. Previous studies have suggested a relationship between productivity and spontaneity in human behavior. One study found that vacuous (non-purpose) objects spontaneously created by the subject stimulated productivity more strongly than objects composed by others (Finke, 1990). In that study, after the object composition, the subjects engaged in a surprise task in which they were required to create novel uses for the objects. Another study engaged subjects in freely editing a Wiki entry by referring to medical documents and found that the number of spontaneous Wiki reconstructions correlated with the degree of knowledge acquisition from the documents, suggesting that, when given the opportunity to use information in spontaneous tasks, subjects will learn the information more strongly (Moskaliuk et al., 2009). Therefore, in the present study, we directly explored the effects of spontaneous selectivity during knowledge acquisition on productivity in an essay composition task.

**Figure 1:**
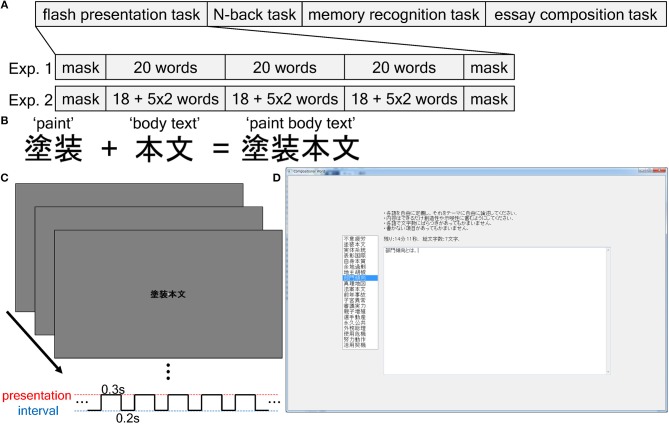
Outline of the present study. **(A)** Task flow. We conducted a flash presentation task, N-back task, memory recognition task, and essay composition task in sequence. In experiment 1, we presented each compositional word once in each block of the flash presentation. Experiment 2 was similar to experiment 1 except that two of the 20 compositional words were presented five times in each block. **(B)** A sample compositional word. Two known Japanese two-character words were combined in a novel manner to yield a novel four-character word. **(C)** The flash presentation task. Compositional words were presented rapidly in the center of a computer display. The duration of one presentation was 300 ms, and the duration of the blank period between the presentations was 200 ms. **(D)** The window application used for the essay composition. The left panel is the box listing the compositional words that appeared in the flash presentation task. The subjects clicked on one of the words in the list box and then wrote an essay on the selected word as creatively as possible in the right text form. After finishing (or aborting) one essay, they selected another word and wrote an essay on the newly selected word. This was repeated for 20 min.

Briefly, we conducted two experiments (Figure 1A). Both experiments consisted of a flash presentation task of novel compositional Japanese words (Figures 1B,C), N-back task, memory recognition task for these words, and essay composition task based on these words (Figure 1D). In experiment 1, we tested whether the acquisition strength of the novel compositional words was positively related to the productivity for the essay composition based on the compositional words. In experiment 2, we examined whether spontaneous selectivity in knowledge acquisition, rather than memory strength *per se*, explained greater productivity. We evaluated the essays from the view of quantity (essay length) as well as the view of quality (semantic distance from components of the compositional word). The results of these experiments suggested that the selective acquisition of knowledge effectively augmented productivity. In addition, we propose a possible mechanism for the observed results based on a neural network simulation of novel information assimilation into a pre-existing schematic knowledge. We show that selective assimilation (corresponding to stronger spontaneous acquisition of words) tended to occur in locations easily accessible to the entire memory network of prior knowledge, which may explain the greater productivity. In addition, we discuss that also the productivity measured from the view of quantity, not only that measured from the view of quality, is creativity-involving.

## Materials and Methods

### Subjects

Thirty-two healthy subjects (11 females; mean age, 21.7 years; age range, 20–25 years) participated in experiment 1, and 30 different healthy subjects (13 females; mean age, 22.4 years; age range, 20–35 years) participated in experiment 2. All subjects were right-handed native Japanese speakers with normal or corrected-to-normal vision. This study and protocol were approved and conducted in accordance with the recommendations of the institutional ethics committee of the National Center of Neurology and Psychiatry. Written informed consent was obtained from all subjects in accordance with the Declaration of Helsinki.

### Experimental Outline

To examine our main hypothesis, we performed two experiments, with each aimed at examining one sub-hypothesis that was derived from the main hypothesis.

#### Experiment 1

In experiment 1, the proposed sub-hypothesis was that the items that the subject more easily acquired constituted richer sources for producing ideas in a text composition task (essay writing based on novel compositional words). Experiment 1 consisted of the following four different tasks: a flash presentation task, N-back working memory task, memory recognition task, and essay composition task (“Exp. 1” in Figure 1A). All were surprise tasks because the subjects were only told that this was a language processing-related experiment, and they did not receive any task-related information in advance. In the flash presentation task, novel compositional words were presented briefly to the subjects on a computer screen. Next, we executed a N-back task to disrupt the short-term memory of the presented compositional words. We then evaluated the memory intensities of the compositional words in a recognition task. Finally, we conducted the essay composition task, in which the subjects were instructed to write essays about the compositional words that were as creative as possible in a limited time. Our main aim was to assess whether the essays that were based on the compositional words that were best remembered according to the recognition test rating were longer (i.e., the words conferred greater productivity) than those that were based on the less remembered words.

#### Experiment 2

In experiment 2, the proposed sub-hypothesis was that selectivity in knowledge acquisition and not memory strength was the reason for the different essay lengths (measure of productivity). To this end, we conducted the same tasks as in experiment 1, except that two compositional words were presented five times in each block to force memorization in the flash presentation task (“Exp. 2” in Figure 1A; see Flash Presentation Task).

### Compositional Words

To create the compositional words, we first extracted the two-character Japanese kanji nouns that appear more than 1,000 times in the corpus of contemporary written Japanese (Maekawa et al., 2014). We then constructed novel compositional words by randomly combining two two-character nouns to form novel four-character kanji words (Figure 1B). In the common Japanese language, the first two characters have an adjectival function over the second two characters. We prepared 20 fixed compositional words (*targets*) that appeared mainly in the three middle blocks of the flash presentation, memory recognition task, and essay composition task. Additionally, we prepared another 20 fixed compositional words (*masks*) that were used in the first and last block of the flash presentation to prevent primacy and recency effects (Murdock, 1962). Such word combinations (or conceptual combinations) are usually considered in creative cognition studies as they require creativity for their interpretation (Wilkenfeld and Ward, 2001; Estes and Ward, 2002).

### Flash Presentation Task

We presented the compositional words sequentially and rapidly at the center of a 15.6-inch (~40 cm) computer display (Figures 1A,C). The distance between the subject and display was ~80 cm. During the task, a white cross was displayed on a gray background in the center of the display as a fixation point. The presentation of each compositional word lasted 0.3 s and was followed by a 0.2-s blank. The task consisted of five blocks. The first and last were masking blocks that were included to prevent primacy and recency effects (Murdock, 1962) and that consisted of 20 mask compositional words. In the second-to-fourth blocks, the 20 target compositional words were shown in each block.

In experiment 1, we presented each word once in each block. In experiment 2, 18 of the 20 words were presented once in each block, and two words selected randomly for each subject were presented five times in each block to force memorization. We minimized the risk of words that were meant to be spontaneously memorized be selected as words to be forcedly memorized by limiting the number of words for forced memorization to two. The presentation order of the words was random within each block. Since each presentation was brief (0.3 s of word presentation and 0.2 s of blank stimulus), the process of pronouncing one word could inadvertently consume attentional resources long enough such as to interfere with perceiving the following word. Therefore, prior to the task, the subjects were instructed to simply watch the words without pronouncing them overtly or covertly. To prevent covert pronunciation, ~70-dB white noise was provided through headphones (USB Headset H390, Logitech Co., Ltd., Tokyo, Japan). The task was performed using Win32 API programming.

### N-Back Task

After the flash presentation of the compositional words, we conducted a two-back task in which two-digit numbers were presented sequentially using the same basic settings as those in the flash presentation task. The duration of each presentation was 0.5 s, and the interval between the numbers was 2.5 s. The subjects were required to respond by pushing the space key within the 0.5-s presentation epoch when the number was the same as the second last one. Correct responses were indicated by the color of the fixation cross changing to magenta, while incorrect responses were indicated by the color of the fixation cross changing to black. In total, 60 numbers were presented over 3 min, and 20 stimuli required responses.

### Memory Recognition Task

In this task, the subjects evaluated their level of confidence that a presented word had appeared during the flash presentation task. Forty compositional words were shown, and 20 were used in the flash presentation task (target words) while 20 were distractors. These distractors were composed by decomposing the target words to their original two-character words and randomly recombining them into four-character compositional words. The subject's confidence was expressed on a scale of 0–100 using sliders on the screen. We instructed the subjects to set the value of the sliders to 100 if they were sure that the word had appeared in the flash presentation task. Alternatively, they were to set the slider to 0 if they were sure that the word had not appeared during the flash presentation task. The middle value (50) corresponded to a middle level of confidence or highest uncertainty. We constructed this task as a window application using the wxPython module (https://wxpython.org/) in Python. Because values < 50 reflected some degree of confidence that the words had not appeared during the flash presentation, they might induce surprise when, in the next task, the subjects were informed that the word had appeared during the flash presentation. Because this was out of the scope of the present study, we limited our analyses to the words for which the level of confidence ranged from 50 to 100.

### Essay Composition Task

We required the subjects to write essays based on the compositional words that were presented in the flash presentation task using a window application that was comprised of a box with the 20 compositional words listed and a text form that was constructed using wxPython (Figure 1D). When the subject selected one of the words by clicking on it, the text form was activated, which enabled them to write an essay on the selected word. When another word was selected by clicking on it, the text form was reactivated, which enabled the subject to write another essay.

The total time provided for the essay composition was 20 min. When the subject clicked on the first compositional word, the countdown started. The remaining time and the current total length of the essays were displayed on the screen. Prior to the task, the subjects were instructed to write the essays as creatively and insightfully as possible, and they were encouraged to select words that would allow them to do that. Therefore, they tended to write the essays about some of the listed compositional words. Indeed, only one subject in experiment 1 wrote essays for all 20 words.

The main factor of interest was the length of the essays, which reflects the productivity conferred by the corresponding words. Since we encouraged subjects to select words which would maximize the creativity and insightfulness of their essays, the essay lengths first depend on their subjective judgments of the ability of certain words to invoke their creative productivity. This is because the essay lengths for the non-selected words were zero. Such judgments are expected to approximately capture the actual ability of the words to invoke creative productivity. Therefore, the length of the essays is considered a primary approximation of measures of productivity in total, especially creativity-involving productivity (see Discussion).

To cancel out the between-subject differences in baseline productivity, we first normalized the length of each essay by dividing it by the total length of all essays for each subject.

Additionally, we semantically analyzed the written essays using a method in natural language processing (see Statistical Analysis).

### Statistical Analysis

In the case of analyzing approximately Gaussian and highly non-Gaussian data, group means and medians were compared by the *t*-test and Mann–Whitney *U*-test, respectively, using the SciPy module (https://www.scipy.org/) in Python. For the independent *t*-test and paired *t*-test, we reported the Cohen's *d* and *d*_*z*_ as the effect sizes, respectively (Lakens, 2013). For the non-parametric test (Mann-Whitney *U*-test), we reported the probability of superiority (*PS*) that is the proportion in which samples in one group are larger than those in the other group (Grissom, 1994) as the effect size. To calculate confidence intervals (CIs), we used the bootstrap method (DiCiccio and Efron, 1996) implemented in the arch module (http://bashtage.github.io/arch/doc/index.html) in Python with the default parameters.

**Figure 2:**
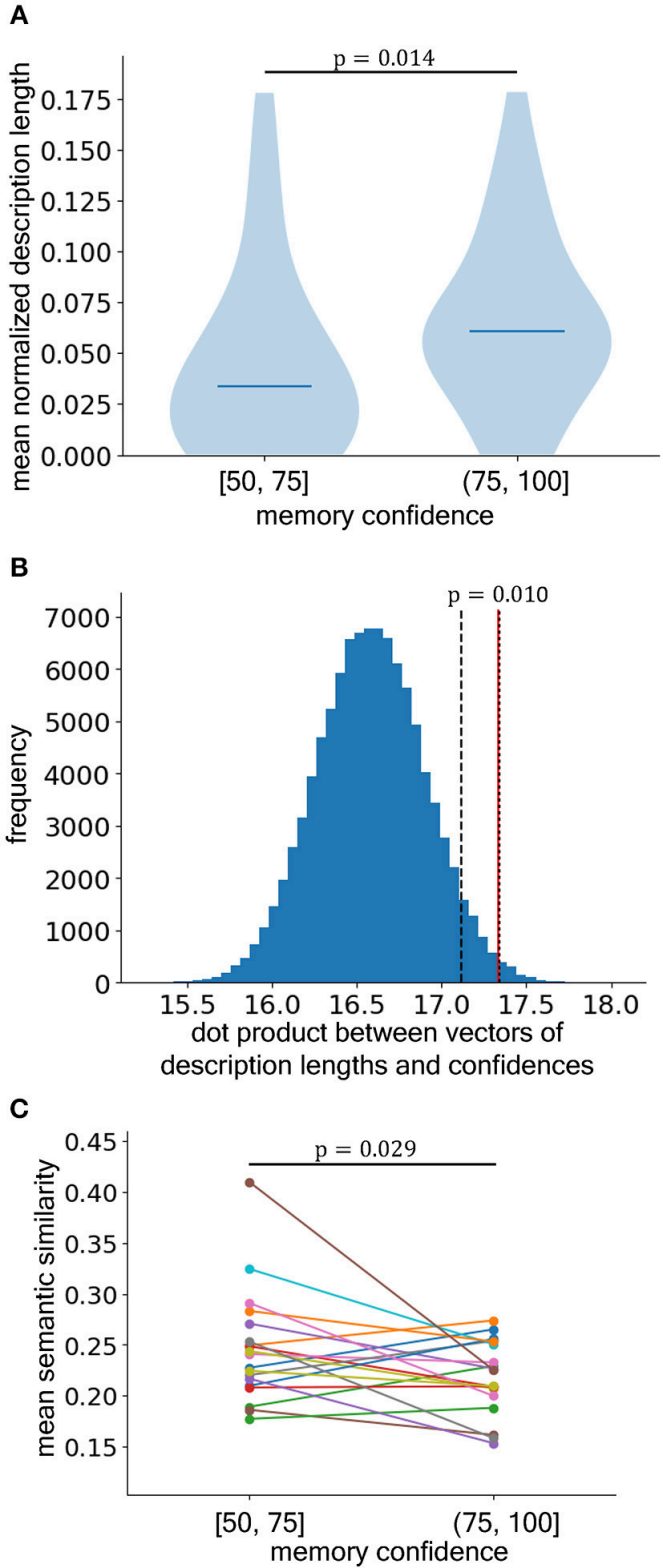
The essays based on the words with higher confidence ratings in the recognition memory task were significantly longer than those based on the low-confidence words. **(A)** The mean description lengths of the essays that were based on the words with lower self-rated confidence that they were included in the flash presentation according to recognition score (left; [50, 75]) and those based on words with higher self-rated confidence [right; (75, 100)] (difference is significant by Mann–Whitney *U*-test, one-sided). The horizontal lines show medians. **(B)** Surrogate data test for the dot product between the unitized vectors of the description lengths and confidence levels. The blue histogram is the distribution sampled from the surrogate data that were generated by shuffling the order of the components in the description length vector. The red line shows the real data. If the level of confidence positively relates with the description length, the location of the redline shifts to the high end of the distribution. The black dashed and dotted lines represent the 5% and 1% significant levels, respectively. **(C)** The semantic similarity between the essay for the compositional word and the components composing the compositional word. Each dot pair connected by line shows the similarities averaged over the compositional words with lower self-rated confidence (left) and those with higher self-rated confidence (right) for each subject (difference is significant by paired *t*-test, one-sided).

**Figure 3:**
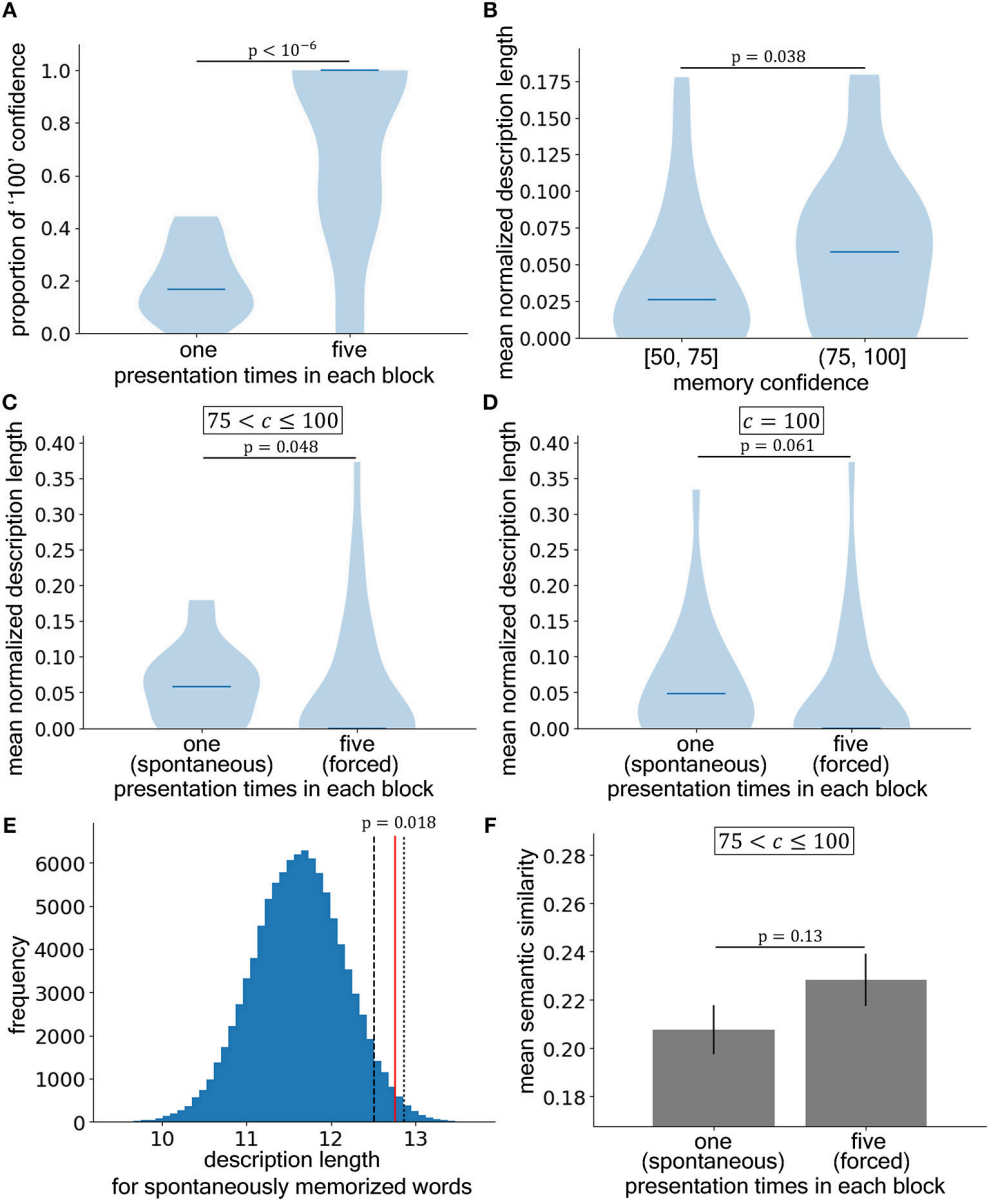
Stronger acquisition *per se* does not explain the higher productivity conferred by spontaneously selected information. **(A)** The proportions of the words judged as absolutely included in the flash presentation (confidence score of 100) in the recognition memory test. The left and right bars correspond to words presented once (spontaneous memorization) and five times (forced memorization), respectively, per block (significant by Mann–Whitney *U*-test, two-sided). **(B)** Comparison of the essay lengths for words presented onetime that were rated with low confidence (left; [50, 75]) or high confidence (right; (75, 100]) of recognition (significant by Mann–Whitney *U*-test, one-sided). **(C)** Comparison of the essay lengths based on spontaneously (left) and forcedly (right) memorized words with high confidence ((75, 100]) (significant by Mann–Whitney *U*-test, one-sided). **(D)** Comparison of the essay lengths based on spontaneously (left) and forcedly (right) memorized words with certain confidence (i.e., 100) (Mann–Whitney *U*-test, one-sided). **(E)** Distribution of the surrogate data from the total normalized description lengths of the spontaneously memorized words. The blue histogram is the distribution of the surrogate data that were generated by reassigning the letters. The red line shows the real data. If the subjects wrote longer essays for the spontaneously memorized words, the location of the red line falls at the higher end of the distribution. The black dashed and dotted lines represent the 5 and 1% significance levels, respectively. **(F)** The semantic similarity between the essay for the compositional word and the components composing the compositional word. The left and right bars correspond to spontaneously (left) and forcedly (right) memorized words, respectively, with high confidence ((75, 100]) (independent *t*-test, one-sided). The horizontal lines in **(A–D)** show the medians.

We also used surrogate data methods in two analyses. First, we tested whether the subjects produced longer essays using the higher confidence words or the lower confidence words (Figure 2B). To this end, we measured the dot product between the unitized vectors of the confidence levels and essay lengths. To produce the surrogate data, we randomly shuffled the order of the components in the vector for essay length and summed them across subjects. The theoretical maximum of the distribution corresponded to the number of subjects, while the minimum corresponded to zero. In this analysis, the value of the dot product increased if the level of confidence was positively related to essay length (i.e., the value was at the high end of the surrogate distribution). Second, we tested whether the subjects produced longer essays using the spontaneously memorized words or the forcedly memorized words (Figure 3E). Here, we considered only the words that were rated 100 for the level of memory confidence (absolute certainty). We normalized the essay lengths again to make the total length equal to 1. We then sampled the surrogate data by randomly reassigning letters to the essays for those words and summed the reassigned lengths of the essays based on the spontaneously memorized words, which yielded the null distribution for the total essay length of the spontaneously memorized words. Because we summed them across subjects, the theoretical maximum corresponded to the number of subjects, while the minimum value corresponded to zero. Again, if the subjects wrote longer essays based on the spontaneously memorized words than those based on the forcedly memorized words, the true value would be at the higher end of the surrogate value distribution. Given that we originally had the one-sided hypotheses (i.e., A is larger than B), as mentioned above, these sub-hypotheses were evaluated with one-sided statistical tests.

Additionally, to more deeply analyze creativity endowed by each compositional word, we measured semantic dissimilarity between the component words composing the compositional word and the essay for the compositional word using the word2vec (Mikolov et al., 2013) implemented in the gensim module (https://radimrehurek.com/gensim/) in Python. We consider that a compositional word that endows more creativity makes leaps of imagination, resulting in increase of the semantic dissimilarity between the component words and the essay. We note that this analysis was applied only to the compositional words that were selected to write by the subjects. Since the subjects was encouraged to select the words enabling them to write essays as creatively as possible, this is considered to analyze “residual” creativity after such a subjective judgment and selection. We constructed the word2vec model using the Japanese Wikipedia (https://ja.wikipedia.org) as a corpus. Generally, words in Japanese text are not separated by spaces. Therefore, first we applied the wp2txt (https://github.com/yohasebe/wp2txt) (Hasebe, 2006) to the tagged Wikipedia texts to extract the plane texts and then we used the MeCab (http://taku910.github.io/mecab/) (Kudo et al., 2004) to make them be space-separated. Then, we executed the algorithm for constructing word2vec model. Using this model, we measured semantic similarity between components of a compositional word and each word appearing in the essay for the compositional word. For each compositional word in the essay, we obtained two similarity values, each of which was correspond to one of two components. Hence, we defined the larger one as the similarity between the word (in the essay) and the compositional word. Then, we averaged similarities for all words in the essay (except same to component words) and defined it as the similarity between the component words and the essay for the compositional word.

### Neural Network Simulation

We constructed a simple and generic neural network model that was composed of *N*_ex_ excitatory and *N*_inh_ inhibitory neurons with their connection strengths governed by biologically plausible synaptic plasticity. The excitatory neuron dynamics were governed by the following differential equation:

$$\begin{array}{l} \tau_{\text{ex}}\frac{dv_{\text{ex},i}}{dt}=-v_{\text{ex},i}+\sum_{j\neq i}w_{\text{ex},4i\leftarrow \text{ex},j}F\left(v_{\text{ex},j}\right)+\sum_{j}w_{\text{ex},i\leftarrow \text{inh},\text{j}}F\left(v_{\text{inh},j}\right)\\ +I_{\text{ex}}. \end{array}$$

Similarly, the inhibitory neuron dynamics were governed by the following equation:

$$\begin{array}{l} \tau_{\text{inh}}\frac{dv_{\text{inh},i}}{dt}=-v_{\text{inh},i}+\sum_{j\neq i}w_{\text{inh},i\leftarrow \text{ex},j}F\left(v_{\text{ex},j}\right)+I_{\text{inh}}. \end{array}$$

In these equations, *v*_ex, *i*_ and *v*_inh, *j*_ express the voltages of the *i*th excitatory neuron and *j*th inhibitory neuron, respectively, and *w*_ex, *i*←ex, *j*_, *w*_ex, *i*←inh, *j*_, and *w*_inh, *i*←ex, *j*_ are the synaptic weights of the excitatory-to-excitatory, inhibitory-to-excitatory, and excitatory-to-inhibitory synapses, respectively. The terms *I*_ex_ and *I*_inh_ are the external noise currents for the excitatory and inhibitory neurons, respectively, and *F*(*v*) is an activation function that is defined by

$$F\left(v\right)=\{\begin{array}{c} v\;(v\geq \Theta )\\ 0\;(v<\Theta ) \end{array},$$

where Θ is the activation threshold. We solved these equations using the fourth-order Runge–Kutta method with a step size of Δ*t*.

Network topology was determined probabilistically. We set the connection probability between excitatory neurons to *P*_ex←ex_ and assumed reciprocal connections which are usually seen in the cortical (Song et al., 2005) and hippocampal CA3 circuits (Guzman et al., 2016). Therefore, we first set half of the connections [i.e., $P_{\text{ex}\leftarrow \text{ex}}\frac{N_{\text{ex}}\left(N_{\text{ex}}-1\right)}{2}$ connections] and then gave them reciprocal connections. Thus, the connection topology matrix was symmetric. The weight matrix was usually nonsymmetrical because each weight varied according to the plasticity rules explained below. The connection probability from excitatory to inhibitory neurons was set to *P*_inh←ex_. Similarly, the connection probability from inhibitory to excitatory neurons was set to *P*_ex← inh_.

In this simulation, the weights of the synapses among excitatory neurons were governed by Hebbian plasticity (Hebb, 1949; Bliss and Collingridge, 1993). We implemented the Bienenstock–Cooper–Munro (BCM) rule, which is a biologically plausible variant of Hebbian plasticity (Bienenstock et al., 1982; Gerstner et al., 2014). The dynamics of the weights were expressed by the following equations:

$$\begin{array}{ll} \tau_{w}\frac{dw_{\text{ex},i\leftarrow \text{ex},j}}{dt}=-w_{\text{ex},i\leftarrow \text{ex},j}+\alpha \phi \left(v_{\text{ex},i},\theta \right)\sigma \left(v_{\text{ex},j}\right)\\ \sigma \left(v_{\text{ex},j}\right)=\frac{1}{1+\exp\left(-\frac{\beta }{v_{\text{ex},j}-\Theta }\right)}\\ \phi \left(v_{\text{ex},i},\theta \right)=\frac{6.75{v_{\text{ex},i}}^{2}\left(v_{\text{ex},i}-\theta \right)}{\theta^{3}}\\ + & \tanh\left(\frac{6.75\left(v_{\text{ex},i}-\theta \right)}{\theta }\right)\\ \theta =\frac{1-\gamma \Delta t}{1-{\left(\gamma t\right)}^{\frac{t}{\Delta t}}}\hat{\theta }\\ \hat{\theta }\leftarrow v_{\text{ex},i}+\gamma \Delta t\hat{\theta }\\ w_{\text{ex},i\leftarrow \text{ex},j}\leftarrow \kappa \frac{w_{\text{ex},i\leftarrow \text{ex},j}}{\sum_{k}w_{\text{ex},i\leftarrow \text{ex},k}}. \end{array}$$

The second term of the first equation expresses the BCM rule as the product of the presynaptic effect σ(*v*_ex, *j*_) and postsynaptic effect ϕ(*v*_ex, *i*_, θ). The presynaptic effect is described by a sigmoid function (the 2nd equation). The third formula defines the postsynaptic effect. It expresses the so-called BCM curve in which above-threshold activity results in synaptic potentiation and below-threshold activity results in synaptic depression. The threshold θ for the BCM curve varied according to the dynamics of the postsynaptic neurons as expressed in the 4th and 5th formulas. The θ becomes the temporal mean voltage of the postsynaptic neuron if the dynamics are stationary. In addition, we implemented synaptic scaling using the last formulation (Turrigiano et al., 1998; Turrigiano, 2008), which prevents infinite divergence of activity caused by a positive feedback loop in which a potentiated synapse induces more potentiation through higher activity in the postsynaptic neuron. Because the dynamics of the synaptic weights were slower than the voltage dynamics, we solved the equation for synaptic weight using the Euler method.

The values of the parameters used in the simulation were as follows: *N*_ex_ = 400, *N*_inh_ = 100, *P*_ex←ex_ = 0.2, *P*_ex←inh_ = 0.2, *P*_inh←ex_ = 0.2, τ_ex_ = τ_inh_ = 20.0 ms, τ_*w*_ = 1000.0 ms, *w*_ex, *i*←inh, *j*_ = −0.025, *w*_inh, i←ex, j_ = 0.2, Θ = 1.0, α = 0.1, β = 5.0, κ = 0.5, and *t* = 0.5 ms. The term *I*_ex_ was drawn from a Gaussian distribution with a mean of 1.0 and standard deviation of 0.5, while *I*_inh_ was drawn from a Gaussian distribution with a mean of 0.0 and standard deviation of 0.5. These variables were calculated following standard numerical methods to integrate the white Gaussian noise (Salinas and Sejnowski, 2002). All units except time were arbitrary.

Using these equations, we performed simulations of information assimilation. After running the simulation until *t* = 50, 000 ms to reach a stationary state, we added a new item to the network. We assumed that each item was represented (encoded) by a triplet of excitatory neurons (three-neuronal cell assembly) (Hebb, 1949; Buzsáki, 2010). Therefore, we added new connections among these three neurons and set the initial values to zero. The simulation was then started again. When *t* = 90, 000 ms, we stopped the simulation. Because network topology was constructed in a probabilistic manner, we ran the simulation with 10 different topologies.

Because we used a recurrent neural network with highly reciprocal connectivity, we assumed that Hebbian learning was induced in cortical horizontal connections but not in Schaffer collateral connections from hippocampal CA3 to CA1 in which plasticity has been widely considered a neural substrate of early memory. However, cumulative evidence suggests that learning without an explicit intention to remember (Atir-Sharon et al., 2015; Merhav et al., 2015) and learning in a way that is highly dependent on pre-existing knowledge (van Kesteren et al., 2010a,b, 2012, 2014; Brod et al., 2016) both bypass the hippocampus, being directly addressed in the cerebral cortex. Since both cases are relevant to our experimental conditions, we consider the network structure in this simulation to be relevant. In addition, many physiological studies, mainly related to NMDA receptor channels, support the BCM rule (Cooper and Bear, 2012), the use of which is therefore acknowledged. In all, on one hand, we consider our model to capture general aspects of pre-existing knowledge-related learning and to fit our experimental condition. However, we note that our assumptions are very simplified and that many aspects of biological neural systems were ignored, including areal interactions, spiking activity, molecular substrates of plasticity, neuromodulators affecting plasticity, and more detailed connectivity properties. Therefore, it is important to stress that this is a highly hypothetical mechanism.

## Results

In the present study, we tested the hypothesis that spontaneously selected knowledge effectively conferred greater productivity. As a task for knowledge acquisition, we presented 20 novel compositional words in a flash presentation task (Figure 1C) and subsequently measured the strength of the acquired memory using subjective ratings from 0 to 100 in a recognition task. To quantify the productivity associated with the compositional words, the subjects produced essays based on the words that were as creative as possible in a limited amount of time (Figure 1D). The individual essay lengths were normalized to the total length of all essays written by the subject, and this value was taken as a measure of productivity conferred by the corresponding words. The main aim of experiment 1 was to examine the relationship between the memory acquisition strength for each compositional word (rating scores in the recognition task) and the length of the corresponding essay. The main aim of experiment 2 was to confirm that such a relationship results from selectivity in knowledge acquisition and not from strong memory *per se*.

### Experiment 1: Positive Relationship Between Knowledge Acquisition and Productivity

The sub-hypothesis tested in experiment 1 was that essay length would increase with memory confidence, which was indicated by the recognition rating. We limited our analyses to words that had confidence levels that ranged from 50 to 100 because scores from 0 to 49 indicated the degree of confidence that the word was not included in the flash presentation. The normalized essay lengths were significantly longer for words with a memory confidence rating *c* in the upper half of the scores (75 < *c* ≤ 100) compared to essays for words with ratings in the lower half (50 ≤ *c* ≤ 75) (Figure 2A), which was consistent with our hypothesis (*U* = 675.5, *p* = 0.014, *n* = 32, *PS* = 0.66, 95% CIs of medians [0.018, 0.047] (50 ≤ *c* ≤ 75) and [0.052, 0.072] (75 < *c* ≤ 100)). Additionally, we conducted an analysis using surrogate data (Figure 2B) and found that the value of the dot product between the confidence level vector and essay length vector from the real data fell at the extreme high end of the shuffled data distribution ($p=0.010,\;n=32,\;n_{\text{surrogate}}=10^{5}$), which again indicated that the level of confidence was positively associated with essay length, thus confirming our first sub-hypothesis.

As we explained (in Essay Composition Task), we encouraged subjects to select compositional words which would maximize the creativity of their essays and then to write the essays. This means that the words selected to write essays were judged as creativity-provoking by the subjects. Therefore, it is assumable that the abilities of selected words to endow creativity-involving productivity are higher than those of the words that were not selected. Since the lengths of essays for the words that the subject did not select were zero, this justifies the mean essay length as a measure of creativity-involving productivity (see also Discussion). On the other hand, the selected words associated with higher memory confidences might more strongly provoke creativity than those associated with lower memory confidences even though both words were judged as creativity-provoking and selected to write creative essays. To investigate such a “residual” difference of ability to endow creativity, we focused on the compositional words that were selected to write essays and evaluated a leap of the imagination using semantic distance between the written essay and the component words that composed the compositional words. If a compositional word endowed more creativity and made leaps of imagination, the written essay for the word should be semantically more distant from the components. Indeed, we found that the essays for words with higher memory confidences were more semantically dissimilar to the components than those for words with lower memory confidences (paired *t*-test, *t*(18) = 2.02, *p* = 0.029, *d*_*z*_ = 0.46, 95% CI of mean difference of semantic similarities [0.0, 0.05]) (Figure 2C). Therefore, even if we focus only on the words subjectively judged as creativity-provoking, this result suggests the positive relationship between knowledge acquisition and creativity-involving productivity.

### Experiment 2: Strong Acquisition of Knowledge *per se* Did Not Explain the Higher Productivity

The results of experiment 1 supported our main hypothesis that spontaneous selectivity in knowledge acquisition results in higher productivity. However, higher productivity might be due to stronger memory *per se* that was indicated by the higher confidence rating. To distinguish between these alternative interpretations, we conducted experiment 2 in which two of the 20 compositional words were presented five times rather than once in each block (Experiment 2 in Figure 1A) to force memorization. Indeed, the frequency of ratings of 100 (absolute certainty that the compositional word was included in the flash presentation) was significantly higher for these two words compared to that for the other 18 words (*U* = 784, *p* < 10^−6^, *n* = 30, *PS* = 0.87, 95% CIs of medians [0.083, 0.22] (*once*) and [1.0, 1.5] (*five times*)) (Figure 3A). In spite of such modification, the essay lengths were still greater for those words that were presented in the same manner as in experiment 1 (once per block) if they were rated in the upper half of the recognition memory score distribution compared to those rated in the lower half of the distribution (*U* = 497, *p* = 0.038, *n*_50 ≤ *c* ≤ 75_ = 26, *n*_75 < *c* ≤ 100_ = 30, *PS* = 0.64, 95% CIs of medians [0.0055, 0.051] (50 ≤ *c* ≤ 75) and [0.034, 0.082] (75 < *c* ≤ 100)) (Figure 3B). Therefore, we were able to reproduce the results of experiment 1.

To test the second sub-hypothesis that selectivity in knowledge acquisition, rather than the difference in memory strength, was the reason for this difference in the lengths of the essays, we focused on the better-memorized words that were in the upper half of the recognition rating distribution (75 < *c* ≤ 100). Henceforth, we refer to the better-memorized words that were presented once per block as *spontaneously memorized words* and the better-memorized words that were presented five times in each block as the *forcedly memorized words*. Consistent with sub-hypothesis 2, the spontaneously memorized words yielded longer essays than the forcedly memorized words (*U* = 522, *p* = 0.048, *n*_spontaneous_ = 30, *n*_forced_ = 28, *PS* = 0.62, 95% CIs of medians [0.034, 0.082] (*spontaneous*) and [−0.046, 0.0] (*forced*)) (Figure 3C).

Moreover, because differences might exist in the confidence level distribution within the range of the upper half (75 < *c* ≤ 100), we compared the lengths of the essays only among the words that were rated with certainty (score of 100). The results were not significant, but they showed a trend that supported sub-hypothesis 2 (*U* = 445, *p* = 0.061, *n*_spontaneous_ = 28, *n*_forced_ = 26, *PS* = 0.61, 95% CIs of medians [0.024, 0.097] (*spontaneous*) and [0.0, 0.0] (*forced*)) (Figure 3D). To further support this sub-hypothesis, we performed a surrogate data analysis in which the letters in the essays were randomly reassigned (Figure 3E), and found that the mean essay length of the spontaneously memorized words fell in the extreme upper part of the surrogate data distribution ($p=0.018,\;n=17,\;n_{\text{surrogate}}=10^{5}$), which was in line with sub-hypothesis 2.

Additionally, we analyzed semantic dissimilarity between the essay for the compositional word selected as creativity-provoking word by the subjects and the component words composing the compositional words. Unlike in the case of Experiment 1, we used the independent *t*-test to test the difference between dissimilarities for spontaneously and forcedly memorized words because the number of subjects who wrote essays for both spontaneously and forcedly memorized words was too small (i.e., *n*_both_ = 7, *n*_spontaneous_ = 22, *n*_forced_ = 9). However, we did not find significant difference (*t*(29) = 1.15, *p* = 0.13, *d* = 0.47, 95% CIs of mean semantic similarities [0.19, 0.23] (*spontaneous*) and [0.21, 0.25] (*forced*)) (Figure 3F). We note that we analyzed residuals after a screening for creativity-provoking words by selecting such words subjectively. Therefore, this result may suggest such a screening well done. In addition, since the effect size is near medium, the sample size (especially for forcedly memorized words) might be too small to detect the residual difference of creativity.

Taken together, these results show that the strong acquisition of the compositional words *per se* does not explain the higher productivity conferred by the better-memorized words. Therefore, we concluded that spontaneous selectivity in knowledge acquisition resulted in greater productivity.

### Simulation: Novel Information That Was Assigned to Locations Easily Accessible to the Entire Network Was Assimilated Better

**Figure 4:**
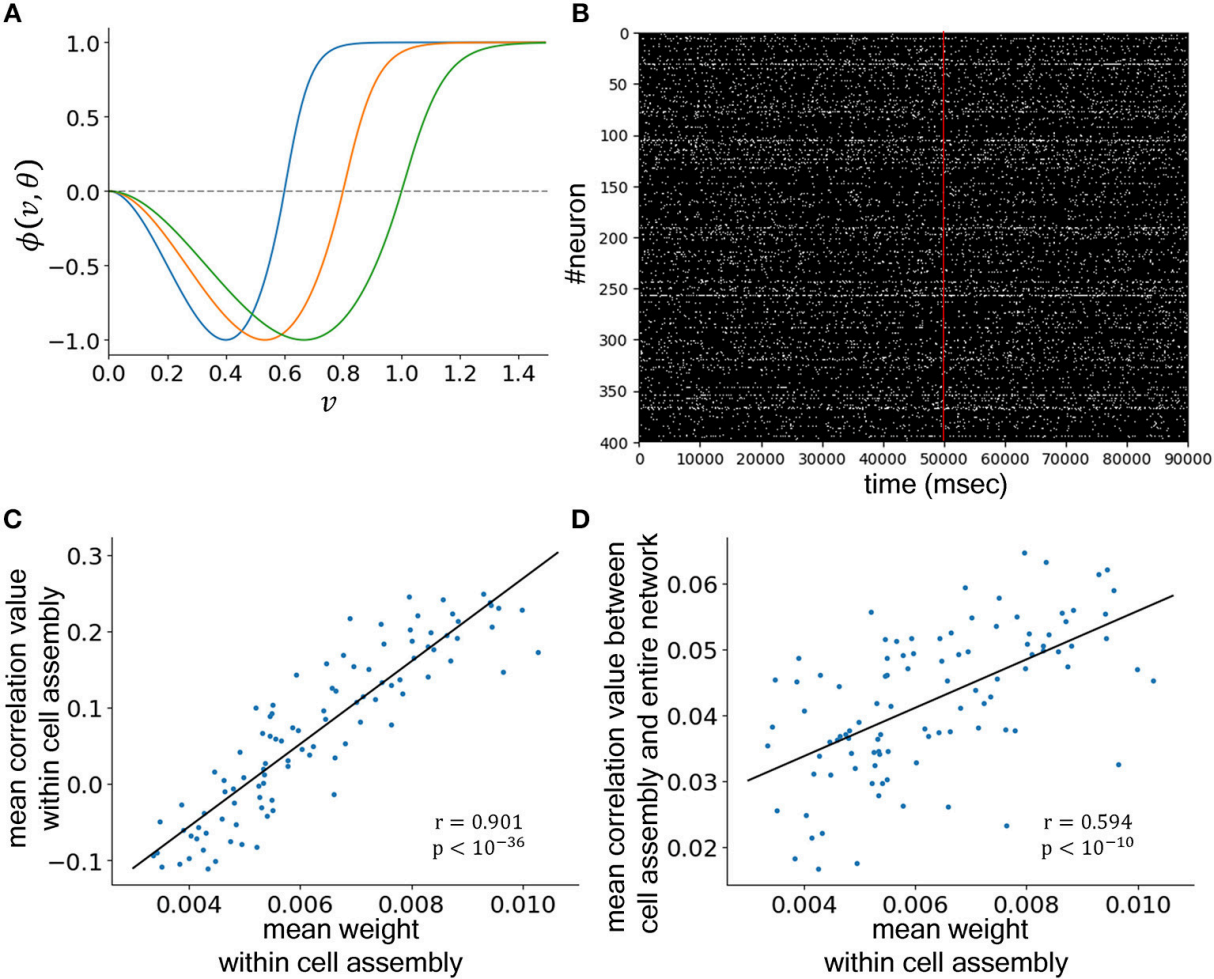
Neural network simulation. **(A)** Bienenstock–Cooper–Munro plasticity curves (direction and magnitude of synaptic strength change vs. neuronal voltage) at different threshold values for the potentiation–depression transition (blue: θ = 0.6, orange: θ = 0.8, green: θ = 1.0). **(B)** Raster plot of excitatory neurons. The activities above 1.0 are plotted in white, while the others are plotted in black. At 50,000 ms (red line), links to make the three-neuronal cell assembly were added. **(C)** Comparison of the mean weights within a cell assembly and the mean correlation values within a cell assembly. **(D)** Comparison of the mean weights within a cell assembly and the mean correlation values between the cell assembly and the entire network. In **(C,D)**, the weight for each link was calculated by averaging the values from 70,000 to 90,000 ms, and the correlation for each neuronal pair was calculated by averaging the values from 30,000 to 50,000 ms.

Considering the results of the two behavioral experiments, we concluded that the selective acquisition of knowledge effectively augmented productivity in the essay composition task. To elucidate the mechanisms, we conducted neural network simulations. We assumed that memories of compositional words encoded through the flash presentation task reflected long-term rather than working memory since they were not washed out by the N-back working memory task and were thus naturally considered to be associated with Hebbian synaptic plasticity. Therefore, we constructed a recurrent neural network model consisting of neurons with reciprocal connections that followed activity-dependent Hebbian and homeostatic plasticity that were regulated by the BCM rule and synaptic scaling, respectively. The BCM curves corresponding to the different threshold values for the potentiation and depression of synaptic strength (θ) are presented in Figure 4A. This network model showed seemingly random baseline activity as is observed in the biological brain (Figure 4B). To model the assignment of new information into the network, we added new connections that linked three neurons to form a cell assembly that was a unit of information representation. For 100 cell assemblies, we compared the strength of assimilation as defined by the synaptic mean weight among these three neurons with the mean activity correlation within the cell assembly and found a strong positive correlation (Figure 4C) across different network topologies (Table 1). This result was not surprising because Hebbian plasticity operates as a detector of correlated activity in pre- and post-synaptic neurons. Moreover, we compared the strength of assimilation to the correlation of activity between the neurons within the cell assembly and neurons outside the cell assembly and found a positive correlation (Figure 4D and Table 1). This result proposed a hypothesis that novel information that was assigned to a cell assembly in a location easily accessible to the entire network tended to be better assimilated.

**Table 1:** Summary of the results of the neural network simulations using 10 different connection topologies.

**Trial**	**Within cell assembly**		**Between cell assembly and entire network**	
	***r***	***p*-value**	***r***	***p*-value**
1	0.847	< 10^−27^	0.442	< 10^−5^
2	0.883	< 10^−33^	0.313	0.0015
3	0.856	< 10^−29^	0.544	< 10^−8^
4	0.860	< 10^−29^	0.353	0.00031
5	0.888	< 10^−34^	0.227	0.023
6	0.880	< 10^−32^	0.323	0.0011
7	0.882	< 10^−33^	0.446	< 10^−5^
8	0.848	< 10^−28^	0.317	0.0013
9	0.846	< 10^−27^	0.203	0.042
10	0.901	< 10^−36^	0.594	< 10^−10^

## Discussion

In the present study, we investigated one consequence of selectivity in knowledge acquisition. If knowledge acquisition is a rational process, the spontaneous selection of information should augment certain cognitive functions. In this study, we hypothesized that selectivity in knowledge acquisition would effectively augment productivity. To test this hypothesis, we conducted two experiments. In experiment 1, we observed a positive relationship between knowledge acquisition and productivity. In experiment 2, we showed that this relationship was not attributable to stronger memory *per se* because strong memories that were acquired spontaneously resulted in greater productivity than strong memories that were induced forcibly. Thus, our main hypothesis was confirmed. Additionally, we conducted neural network simulations to explore the mechanisms underlying the experimental results. The results of this simulation suggested that the selective assimilation of information that was assigned to a location that was easily accessible to the entire network resulted in greater productivity.

We note that most of what we found and reported in this study is that spontaneously selected words result in the production of longer essays in the time-limited, creativity-contingent text composition task. Although many factors may affect the length of the essays (e.g., provoked thoughts, associability with pre-existing knowledge, and individual essay writing strategies), they are expected, since subjects were encouraged to pick words that would maximize the creativity and insightfulness of their essays, to be mainly dependent on individuals' subjective judgments of the ability of a given compositional word to invoke their creative productivity. Therefore, the essay lengths mainly reflect a subject's rating of a word's ability to generate creative productivity (since the essay lengths for non-selected words were zero). Such a rating is expected to approximately capture actual generation of creative productivity. Of course, also an actual ability of word to endow productivity is directly reflected in the essay length. We thus consider the essay lengths to be the primary approximation of measures of total productivity that is at least partially creativity-involving. In addition, though we cannot exclude the possibility that subjects had the self-motivation to write longer essays, it is important to note that we did not encourage it. This likely means that subjects tapped into their subjectively-fueled creative productivity.

Although the compositional words that the subjects selected and wrote the essays for were judged as creativity-provoking by them, the semantic analysis revealed difference of creativity between the essays for the words whose memory confidence were higher and lower even after such judgments. Therefore, the positive relationship between selective knowledge acquisition and creativity-involving productivity is suggested not only by the quantitative measure (i.e., essay length) but also by qualitative measure (i.e., semantic dissimilarity). On the other hand, we did not find significant difference between semantically-measured creativity in the essays for the spontaneously memorized words and that in the essays for the forcedly memorized words. However, we note again that this analysis was applied only to the essays for the words judged as creativity-provoking and selected by the subjects. Therefore, we consider that the difference of abilities to endow creativity between those words was small compared to that between the selected and non-selected words. In addition, especially for forcedly memorized words, the number of the words that the subjects actually wrote the essays for might be too small to detect such a residual difference, which is suggested by not-so-small effect size.

### Interpretations of the Experimental Results

We demonstrated that spontaneous selectivity in knowledge acquisition resulted in the effective augmentation of productivity. Although our simulations suggested that biological plasticity rules might be the link between selectivity and productivity, we are open to any interpretation of the underlying mechanisms because our behavioral experiments alone did not specify the actual causality and factors latent in the observed link.

We considered at least four non-exclusive interpretations of the observed results. The first was that a latent common factor governed both knowledge acquisition and high productivity, such as the degree of associability with pre-existing schematic knowledge. Novel information might be acquired better if the information is assigned to a location that allows it to associate with the majority of the pre-existing knowledge. This location would also allow the information to activate the majority of the knowledge more readily, thus resulting in greater productivity. Thus, novel information that tends to be acquired better (i.e., more confidently recognized) might effectively augment productivity (i.e., longer essays) because it activates more pre-existing knowledge. Our simulation that implemented Hebbian plasticity suggested this possibility. Alternatively, novel information that associates with a more richly organized subdomain of knowledge might be acquired better. In this case, the novel information that is acquired better can be considered a conduit to more productive knowledge, which results in greater productivity. These mechanisms are in accord with previous studies reporting the facilitation of knowledge acquisition by pre-existing knowledge (van Kesteren et al., 2010a,b, 2013, 2014; van Buuren et al., 2014; Brod et al., 2015, 2016; Liu et al., 2017; Sommer, 2017). To investigate such mechanisms further, we may need to infer individual pre-existing schematic knowledge and reveal relationships between the knowledge structure, selectivity in knowledge acquisition, and productivity based on newly acquired knowledge. In order to infer the structure of individual knowledge, we plan on applying a representational similarity analysis (Kriegeskorte et al., 2008; Carota et al., 2017) on fMRI signals activated using large-scale naturalistic stimuli (Huth et al., 2012, 2016) as well as conducting a free association task (Kenett et al., 2014, 2018) for the construction of an individual semantic network.

The second interpretation was that humans actively select information that augments productivity effectively. In other words, knowledge is selectively acquired with the gradient of productivity as a direct driving force. This is one form of the optimization-theoretical view of cognitive processing in which our cognitive acts are governed by objective functions (optimization functions). In this study, productivity was an example of an objective function, with the resultant lengths of the essays corresponding to the function's return values (or differences in return values). Therefore, the expected increment of productivity conferred by the information determined the degree of acquisition selectivity. Thus, the spontaneous selection of information might directly increase productivity. With this view, the augmentation of productivity is considered a *cause* rather than a *consequence* because it defines an effective force that drives selective knowledge acquisition. We straightforwardly consider that this force is related to the motivation to produce. Therefore, to test this interpretation, we can control the motivation (i.e., gradient of productivity) through controlling reward or opportunity for production based on newly acquired knowledge and observe the consequent effect on selectivity. In addition, we can test this interpretation (i.e., heightened productivity as a cause and selectivity in knowledge acquisition as a consequence) by inferring individual motivation and applying causal inference methods based on non-Gaussianity (Shimizu et al., 2006; Shimizu and Kano, 2008) into paired data on motivation and selectivity. If this interpretation is true, however, we need to note that productivity is probably not the only objective function. By definition, the values of objective functions governing knowledge acquisition necessarily increase along with the spontaneous acquisition of information. Considering this criterion, predictability may be an objective function because it is usually augmented by the acquisition of knowledge. Several theoretical studies have proposed that an increase in predictability is a more general principle that governs the dynamics of the brain (Bar, 2007, 2009, Friston, 2005, 2009, 2010). Therefore, we may need to consider knowledge acquisition with respect of not only productivity augmentation but also predictability augmentation.

The third interpretation was that the spontaneous selection of knowledge alone augmented productivity. For this view, the selection of information also likely directly increased productivity. We are open to this possibility. However, this does not mean that an item resulting in higher productivity was decided randomly. As discussed in the Introduction, knowledge acquisition is characterized by selectivity, which is governed by several cognitive factors (e.g., congruency with pre-existing schematic knowledge, curiosity, and anticipation of use) that can be evaluated based on subjective ratings and investigation of individual semantic networks (see above). Therefore, for this interpretation, signatures characterizing the spontaneous selection of knowledge also indirectly characterize the item endowing higher productivity. This view implies a path diagram (causal network) from cognitive factors to productivity via spontaneous knowledge acquisition. Since productivity becomes independent of the cognitive factors under conditioning with spontaneous selectivity in knowledge acquisition, we can test this interpretation based on conditional independence.

The fourth interpretation was that the attention to an item caused both stronger acquisition and greater productivity. In other words, attention is considered a medium that links them. In this case, items attracting attention are not decided randomly because of the characteristic selectivity in knowledge acquisition. Although attention enhances memory acquisition (Moray, 1959), the relationship between attention and productivity is unclear. Therefore, to confirm this interpretation, we need to elucidate causality between attention and productivity. We can control the bottom-up attentional strength by changing the brightness of the presentations of compositional words and gauge top-down attentional strength from pupil size (Hoeks and Levelt, 1993) and electrodermal activity (Raskin, 1973).

All four interpretations lead us to consider selective knowledge acquisition as an augmentation process of creativity-involving productivity. This means that through selectivity in knowledge acquisition, the brain automatically acquires information that will be a useful resource for subsequent production. This view is justified regardless of whether a gradient of productivity directly drives the selectivity in knowledge acquisition.

As stated above, these four interpretations are non-exclusive. In addition, it is possible that productivity augmentation is achieved through a combination of mechanisms, each utilizing different sources for the productivity. In the first, the source for the productivity is prior knowledge, and the new information effectively provokes its recall. In the second, the newly assimilated information *per se* is the resource for productivity. In the third, the source emerges *de novo* through an interaction of the prior knowledge with the new information. In the present study, the interpretations as well as the sources of productivity are entangled. Therefore, more sophisticated experiments are necessary to isolate them.

Although we did not inform about essay composition task before presenting novel compositional words, the subjects might expect to be rewarded for acquisition of creative productivity-invoking items from their everyday lives. In this case, our observations are possibly reduced to reward-based reinforcement learning algorithms (Sutton and Barto, 1998). On the other hand, the subjects might engage in more spontaneous learning to update their internal models for the world. Since the compositional words were novel concepts possibly elucidating certain aspects of the world, they should drive the subjects to update their internal models. The higher creativity-invoking productivity endowed by spontaneously acquired words might reflect large differences between prior and updated models. From a computational view, this may be reasonable because the driving force governing such spontaneous learning is modeled as a gradient of (pseudo-)distance between prior and updated models (e.g., Kullback-Leibler divergence) (Storck et al., 1995; Baldi and Itti, 2005; Itti and Baldi, 2006; Little and Sommer, 2013).

### Implications of the Present Study

Several factors appear to influence selectivity in knowledge acquisition, including prior knowledge, familiarity, anticipation of utility, and natural curiosity. Furthermore, brain areas related to selective knowledge acquisition have been identified. However, little is known about the functionality of such selectivity. To understand this functionality, it is necessary to understand the consequences of the selectivity as well as the principles governing the selectivity, which roughly correspond to the highest (computational theory) of Marr's three levels of understanding of information processing systems (Marr, 1982). Our experiments identified one such consequence (augmentation of productivity). In other words, the augmentation of productivity can be considered one of the goals of selective knowledge acquisition. Additionally, as we mentioned in the section above on the interpretations of the experimental results, our observations moderately suggest the existence of an optimization process in which productivity is the objective function latent in selectivity. Moreover, our neural network simulation showed that the newly selected acquired information was encoded in a location that was easily accessible to the entire network when the connectivity was governed according to Hebbian and homeostatic plasticity rules. Because each neuron or cell assembly can be considered to represent information in the knowledge schema, this means that information is well-acquired when it is effectively linked to the entire body of knowledge. We suggest that this is a possible mechanism underlying not only the augmentation of productivity but also the facilitated learning of items that are congruent with prior knowledge (van Kesteren et al., 2010a,b, 2013, 2014; van Buuren et al., 2014; Brod et al., 2015, 2016; Liu et al., 2017; Sommer, 2017).

Our observations shed light on the proactive nature of the brain because selectivity in knowledge acquisition contributes to future productivity. As a result, the brain can be considered to already ‘know’ what items will enhance future productivity, at least at the stage of knowledge acquisition. Elucidating the precise neural mechanisms enabling such proactive knowledge acquisition requires much further study. Our simulation suggested some involvement of Hebbian and homeostatic plasticity. Identifying when the brain decides that certain information is important to acquire is an essential first step. An electroencephalography study has suggested that this may occur shortly after presentation (within a few hundred milliseconds) (Packard et al., 2017). A recent study that showed that pupil dilation occurred 1 s after the presentation of an item predicted that successful encoding may be additional evidence of this view (Kucewicz et al., 2018).

In the present study, we used a creativity-demanding task and showed the association between selective knowledge acquisition and creativity-involving productivity. Although we do not allege that we distilled pure creativity, we consider that our results provide insights for creativity research. Because we instructed the subjects to write their essays as creatively as possible, they did engage in the task creatively in some naturalistic sense using their subjective judgments. As we discussed above, therefore, the productivity measured in this study (essay length) is expected to be creativity-involving. If it actually reflects creativity that is conferred by acquired item, the most important insight is that knowledge acquisition may automatically lead to the augmentation of creativity. This is compatible with the notion that one's creativity positively and monotonically relates to the degree of expertise (Weisberg, 1999, 2006) while it appears to contradict the view that knowledge may confer tunnel vision (Frensch and Sternberg, 1989). Another possible insight from the results of the present study is that information conferring greater creativity may be assigned to neural network locations that are more easily accessible to the entire network. Applying a percolation analysis to a semantic network that was constructed based on free association task data showed that the semantic networks that were possessed by highly creative people had robustness to removal of the edges (Kenett et al., 2018). The nodes in such semantic networks were highly accessible to each other. Therefore, while that study illustrated a close relationship between global accessibility among all network nodes and creativity, the simulations conducted in the present study suggested a relationship between the accessibility of an item to the entire network and the creativity conferred by that item.

### Limitations and Future Directions

In our experimental design, we encouraged the subjects to select words that would allow them to write the most creative and insightful essays. Therefore, they tended to write essays about only certain words, and they subjectively judged whether a given word would enhance their creative productivity. We accounted for these subjective judgements, but we believe that they were highly correlated with the actual productivity. In natural situations, we probably choose themes that allow for the greatest individual productivity when we are required to be productive. If so, then the resulting productivity is largely determined by the theme selection. Therefore, our measurement of productivity with essay lengths was thought to capture productivity in natural scenes and/or in totality. However, in future studies, it will be important to distinguish subjective judgments from actual productivity and analyze the tripartite relationships among the subjective judgments of productivity, actual productivity, and selectivity in knowledge acquisition. In addition, we note that our measure (lengths of essays), since confounded by factors such as contingent spotting of some words and individual word picking and essay writing strategies, was not purely reflective of productivity. These confounding factors partially resulted from the time limited essay composition task. Moreover, due to this time limit, nearly no subject completed the essay composition for all compositional words. We therefore could only apply incomplete analysis of essay qualities to each compositional word. To address this, we plan on conducting another experiment in which the essay composition task will be conducted without a time limit. In the experiment, the essays will be analyzed from various perspectives (e.g., subjective and third person's evaluations using adjective rating scales and more sophisticated natural language processing methods to show syntactic and semantic features of the essays) to more clearly evaluate the consequences of selectivity in knowledge acquisition. In addition, it is important to utilize multidimensional measurements (not only essay composition) for each compositional word including free associations with the word.

In the present study, we identified the augmentation of productivity as one consequence of spontaneous selectivity in knowledge acquisition. However, this is probably not the only consequence. As mentioned in section above on the interpretations of the experimental results, the augmentation of predictability may be another consequence of selectivity in knowledge acquisition. Because we believe that there may be dozens of consequences resulting from selectivity in knowledge acquisition, it is necessary to explore these exhaustively. Moreover, we should be able to identify actual logical causality between the consequences and selectivity in knowledge acquisition. In the section above on the interpretations of the experimental results, we argue for the possibility that productivity can be a cause of selectivity if it is given as an objective function in the optimization process. Therefore, to understand how knowledge acquisition determines our future knowledge, worldviews, and entire cognitive features, we need to exhaustively disentangle the principles, causes, and consequences of selectivity. We must elucidate the entire relational network that is centered on selectivity in knowledge acquisition, which includes an explanation of our present observations. Such an exhaustive exploration is the most important future direction.

We navigated various experimental parameters, including the number of compositional words, the time and duration of flash presentations of the words, and the time allocated for essay writing. The number of forcedly memorized words may be an issue. As mentioned, in the section Flash Presentation Task, we decided to minimize the risk that some words to be spontaneously memorized be selected as words to be forcedly memorized. With regards to generating enough statistical power, two words selected for this intent could be too considered too little. As such, it is important to note that the increase in the number of forcedly memorized words does not necessarily increase power since the test's sample size is not the number of words, but the number of subjects. Although increase of the number of forcedly memorized words may affect power by changing the effect sizes due to change in population variances for spontaneously and forcedly memorized words, this dynamic is not known. In the analysis of semantic dissimilarity, our choice of the number of forcedly memorized words might lead to small power. However, this was caused also by subjects' selections of the word to write. Therefore, this problem should be resolved using more sophisticated experimental protocol as we mentioned above.

Several researchers have recently suggested that conventionally used significance levels (α = 0.05) are not conservative enough to support the replicability of scientific studies, including those within psychology and cognitive neuroscience, and have proposed to redefine the significance level as 0.005 (Benjamin et al., 2018). In this study, since we used a conventional significance level to test our hypotheses, our statistical analysis may not be robust enough. However, it is important to stress that the result of experiment 1 were qualitatively replicated in the experiment 2. Therefore, we conclude that the first sub-hypothesis has strong reason to be supported. The second sub-hypothesis, however, requires more evidence.

The neural network model addressed in the present study had recurrent connections which are widely observed in the cerebral cortex. In addition, we used a biologically plausible model of Hebbian plasticity. Such simple assumptions make the results general. Nonetheless, since we ignored many biological details, this model is only a hypothetical candidate for explaining our experimental observations.

## Conclusion

In the present study, we identified effective augmentation of productivity that is at least partially creativity-involving as one *consequence* of selectivity in knowledge acquisition. Thus, we now consider selective knowledge acquisition as an augmentation process of the creativity-involving productivity. Moreover, on the basis of the neural network simulation, we proposed a hypothesis that the selective assimilation of information assigned to a location that is easily accessible to the entire neural network is a mechanism underlying the augmentation of productivity. The results of this study provide significant insights for how selective knowledge acquisition sculpts our entire cognition.

## Data Availability

The datasets for this manuscript are not publicly available because the protocol approved by the institutional ethics committee did not include publication of the original datasets. Requests to access the datasets should be directed to HK (h.kura00@gmail.com).

## Author Contributions

HK performed the experiments and data analysis. All authors contributed to the design of the study and writing of the manuscript.

### Conflict of Interest Statement

The authors declare that the research was conducted in the absence of any commercial or financial relationships that could be construed as a potential conflict of interest.
